# Active and passive smoking and the risk of breast cancer in women aged 36–45 years: a population based case–control study in the UK

**DOI:** 10.1038/sj.bjc.6603859

**Published:** 2007-06-19

**Authors:** A W Roddam, K Pirie, M C Pike, C Chilvers, B Crossley, C Hermon, K McPherson, J Peto, M Vessey, V Beral

**Affiliations:** 1 Cancer Research UK Epidemiology Unit, Richard Doll Building, University of Oxford, Old Road Campus, Headington, Oxford OX3 7LF, UK; 2 Department of Preventive Medicine, Norris Comprehensive Cancer Center, Keck School of Medicine, University of Southern California, Los Angeles, CA 90033, USA; 3 Department of Health, E Floor, Government Office for the East Midlands, The Belgrave Centre, Stanley Place, Talbot Street, Nottingham, NG1 5GG, UK; 4 Nuffield Department of Obstetrics and Gynaecology, University of Oxford, Womens Centre, Level 3, John Radcliffe Hospital, Headington, Oxford OX3 9DU, UK; 5 Cancer Research UK Section of Epidemiology, Brookes Lawley Building, Institute of Cancer Research, Sutton, Surrey SM2 5NG, UK; 6 Department of Epidemiology and Population Health, London School of Hygiene and Tropical Medicine, Keppel Street, London WC1E 7HT, UK; 7 Unit of Health Care Epidemiology, Department of Public Health, Old Road Campus, Headington, Oxford OX3 7LF, UK

**Keywords:** breast cancer, smoking, passive smoking, case-control study

## Abstract

Active smoking has little or no effect on breast cancer risk but some investigators have suggested that passive smoking and its interaction with active smoking may be associated with an increased risk. In a population based case–control study of breast cancer in women aged 36–45 years at diagnosis, information on active smoking, passive smoking in the home, and other factors, was collected at interview from 639 cases and 640 controls. Women were categorised jointly by their active and passive smoking exposure. Among never smoking controls, women who also reported no passive smoking exposure were significantly more likely to be nulliparous and to be recent users of oral contraceptives. Among those never exposed to passive smoking, there was no significant association between active smoking and breast cancer, relative risk (RR) of 1.12 (95% confidence interval (CI) 0.72–1.73) for past smokers and RR of 1.19 (95% CI 0.72–1.95) for current smokers, nor was there an association with age started, duration or intensity of active smoking. Compared with women who were never active nor passive smokers, there was no significant association between passive smoking in the home and breast cancer risk in never smokers, RR of 0.89 (95% CI 0.64–1.25), in past smokers, RR of 1.09 (95% CI 0.75–1.56), or in current smokers, RR of 0.93 (95% CI 0.67–1.30). There was no trend with increasing duration of passive smoking and there was no heterogeneity among any of the subgroups examined. In this study, there was no evidence of an association between either active smoking or passive smoking in the home and risk of breast cancer.

Active smoking has little or no effect on a woman's risk of developing breast cancer (Collaborative Group on Hormonal Factors in Breast Cancer, 2002a; IARC, 2004), although there remains some uncertainty over whether or not there is an effect with long duration of use (Terry and Rohan, 2002). There is considerable controversy over the possible association between passive smoking and breast cancer risk, with much uncertainty over whether any association may be stronger for premenopausal compared to postmenopausal women (Johnson, 2005; US Surgeon General, 2006; Miller *et al*, 2007). Furthermore, it has been suggested that the lack of an effect of active smoking on breast cancer risk might be due to the reference group of never smokers being confounded by passive smokers (Johnson, 2005).

We report here on the relationship between exposure to active smoking and passive smoking within the home and breast cancer risk in women aged 36–45 years using data from a UK population based case–control study which was designed to investigate the relationship between oral contraceptive use and breast cancer risk, and also collected data on a range of lifestyle factors including active and passive smoking history.

## MATERIALS AND METHODS

### Study population

This is a population based case–control study of breast cancer that recruited women who were residents of the Thames, Oxford and Yorkshire regions, aged 36–45 years who were diagnosed between July 1987 and February 1990. The study was restricted to white women with no previous malignancy (except non-melanoma skin cancer), severe mental handicap or significant illness.

Cases were women with an incident invasive breast cancer, identified chiefly through the Regional Cancer Registries, supplemented by hospital discharge records from Hospital Activity Analysis registers and patient lists kept at major treatment centres. These latter two methods were used to help minimise the delay between diagnosis and interview. All cases were confirmed by cancer registration. Permission from hospital consultants and general practitioners was sought before the cases’ identities were disclosed from the Cancer Registry and before the women were contacted. Breast cancer patients’ case-notes were reviewed, confirming the diagnosis in 97% of the cases. Out of 838 eligible cases, 644 (77%) were interviewed. Reasons for nonparticipation were consultants or general practitioners’ refusal (6%), woman's own refusal (3%), death (11%) and relocation from the study area (3%).

For every participating case, one age-matched control was randomly selected from patients registered with the case's general practitioner. If a chosen control could not be interviewed, a further age-matched control was selected. Of the 644 first eligible controls, 588 (91%) were interviewed. Reasons for nonparticipation of the primarily selected controls were general practitioners’ refusal (3%) or woman's own refusal (6%).

### Data collection

Study participants were interviewed in their homes, using a standardised structured questionnaire. Each case–control pair was interviewed by the same, trained, interviewer. The mean time between diagnosis and interview was approximately 2 years (range 1–5 years). Women were asked whether they had ever smoked, and if so, how old they were when they started. For each subsequent year, women were asked to report the number of months of smoking and the average number of cigarettes smoked per day (including manufactured/hand-rolled cigarettes and small cigars). In addition, the participants were asked to report for each year from the age of 16 years if they were married or living with a boyfriend, and if yes how many cigarettes per day he smoked at home (none, 1–4, 5–14, 15–24, 25+). In addition, information was sought from each woman about her menstrual and reproductive history and other risk factors for breast cancer.

### Active and passive smoking exposure

Women were defined as current smokers if they reported being a smoker in the year prior to interview, and as past smokers if they had stopped smoking for at least 1 year prior to interview. Women were categorised as being passive smokers if they reported living for at least 1 year with a partner who smoked at home. Among women who were passive smokers, the total number of years of exposure and the cigarette-years of exposure were calculated, the latter as the number of years of exposure multiplied by the number of cigarettes smoked per day in the home.

### Statistical analyses

Women were defined as peri/postmenopausal if they reported having had a natural menopause (*n*=14), bilateral oophorectomy (*n*=6) or were users of hormone replacement therapy (*n*=73). One control who did not have information on active smoking exposure along with three controls and five cases who did not have information on passive smoking exposure were excluded leaving 639 cases and 640 controls for analysis.

Differences in characteristics were assessed using either *χ*^2^ tests for categorical variables or *t*-tests for continuous variables. Odds ratios as estimates of relative risks (RRs) and 95% confidence intervals (CIs) were calculated using conditional logistic regression with stratification by age at diagnosis/pseudodiagnosis (single year) and region of recruitment. All estimates are presented stratified by age and region and additionally adjusted for socioeconomic status of the woman (according to the Registrar Generals classification: professional (SC1 or SC2), nonmanual (SC3), manual/not employed (SC4/SC5/other)), alcohol consumption (non-drinkers, 1–50 g week^−1^, 50+ g week^−1^), BMI (<22 kg m^−2^, 22–25 kg m^−2^, 25+ kg m^−2^), parity and age at first giving birth (nulliparous, parous and age at first giving birth <25 years, parous and age at first giving birth ⩾25 years), use of oral contraceptives (never user, ever user within the last 5 years, ever user and last use more than 5 years ago), family history of breast cancer (no history, mother and/or sister with breast cancer), age at menarche (<13 and 13+ years) and menopausal status (premenopausal, peri/postmenopausal). These adjustments were included on the basis of *a priori* evidence of their effects on breast cancer risk irrespective of their significance in this study. The effect of passive smoking exposure was examined in a number of subgroups known to be related to breast cancer risk – menopausal status, alcohol consumption, oral contraceptive use, family history of breast cancer, parity and age at first birth, socioeconomic status, BMI and age at menarche. In these analyses, RRs were estimated from conditional logistic regression models stratified by age and region and adjusted for parity (nulliparous, parous) and oral contraceptive use (never user, last use within 5 years, last use 5+ years) where appropriate. Heterogeneities between estimates of RRs were assessed using *χ*^2^ tests.

All analyses were performed in Stata version 8.1 (Stata Corporation, College Station, TX, USA). All statistical tests were two sided, and *P*-values less than 0.05 were regarded as statistically significant.

## RESULTS

Characteristics of the cases and controls included in these analyses are presented in Table 1. Cases and controls were of a similar socioeconomic class, had a similar pattern of alcohol consumption, history of oral contraceptive use, parity and menopausal status. Cases were more likely to have a family history of breast cancer and to have a lower mean BMI. Mean age at menarche and mean age at first giving birth among parous women was similar between cases and controls.

Among participants, 300 (23%) reported being a past smoker and 372 (29%) reported being a current smoker, and a higher proportion of ever smokers also reported exposure to passive smoking in the home (42, 63 and 78% among never, past and current smokers, respectively). Similar rates of exposure to passive smoking in the home by case and control status were reported for never smokers (41 and 44% in cases and controls, respectively) and ever smokers (70 and 73% in cases and controls, respectively). Among smokers, the average number of cigarettes per day, duration of smoking and age started smoking were similar between cases and controls. The duration of exposure to passive smoking was similar between cases and controls for never, past and current smokers.

Among controls who reported never smoking, there were no significant differences between those with, and those without, exposure to passive smoking and socioeconomic status, family history of breast cancer, BMI, age at menarche, menopausal status and age at first giving birth (Table 2). There was also no difference in their mean alcohol consumption; women exposed to passive smoking consumed 4.3 g day^−1^ compared with 4.4 g day^−1^ in women not exposed. However, there were significant differences between exposure to passive smoking and both parity and oral contraceptive use (*P*<0.05, Table 2). Compared with women who reported exposure to passive smoking, those who were not exposed were significantly more likely to be nulliparous, and to be either never or recent users of oral contraceptives.

There was no association between active smoking and breast cancer risk with an RR of 1.15 (95% CI 0.87–1.53) for past smokers and an RR of 1.04 (95% CI 0.79–1.36) for current smokers compared with never smokers (Table 3). Similarly, there was no association between smoking intensity, duration of smoking and age started smoking and breast cancer risk either individually or after mutual adjustment (Table 3). Among those participants never exposed to passive smoking, there was no association between past or current smoking and breast cancer risk with an RR of 1.12 (95% CI 0.72–1.73) for past smokers and an RR of 1.19 (95% CI 0.72–1.95) for current smokers. Compared with women who were never active smokers nor exposed to passive smoking, there was no significant association between exposure to passive smoking in the home and breast cancer risk in never smokers with an RR of 0.89 (95% CI 0.64–1.25), in past smokers with an RR of 1.09 (95% CI 0.75–1.56) or in current smokers with an RR of 0.93 (95% CI 0.67–1.30). There was no significant trend between the number of years of exposure to passive smoking and breast cancer risk by active smoking exposure (Table 3).

Among the never smokers, there was no significant heterogeneity between risk of breast cancer and exposure to passive smoking for a range of participant characteristics (Table 4). The association between passive smoking and breast cancer risk was consistent in subgroups of menopausal status (although 93% were premenopausal), alcohol consumption, oral contraceptive use, family history of breast cancer, parity and age at first giving birth, socioeconomic status, BMI and age at menarche.

## DISCUSSION

In this study, neither being an active smoker nor exposure to passive smoking in the home from a partner was associated with breast cancer risk among women aged 36–45 years. There was no association between smoking intensity, duration or age started smoking and breast cancer risk, neither was there a trend with increasing duration of exposure of passive smoking. Adjustment for established breast cancer risk factors made little difference to risk estimates, and the lack of association between passive smoking and breast cancer risk was consistent among a number of subgroups including menopausal status, alcohol consumption, oral contraceptive use, family history of breast cancer, parity and age at first giving birth, socioeconomic status, BMI and age at menarche.

The lack of an association between active smoking and breast cancer risk in the current study is consistent with previous reviews and a collaborative reanalysis of worldwide data (Collaborative Group on Hormonal Factors in Breast Cancer, 2002a; Terry and Rohan, 2002; IARC, 2004; US Surgeon General, 2006). Contrary to some hypotheses (Terry and Rohan, 2002), the current study found no evidence either of an association with increasing intensity or duration of exposure to tobacco smoke, nor is there evidence of a relationship between age at initiation of smoking and breast cancer risk. Furthermore, it has been suggested that this lack of an association may be due to the reference group of never smokers being confounded by passive smoking exposure (Johnson, 2005). In the current analysis, we investigated this question by setting the reference group to be never smokers who had also reported no passive smoking exposure and found there to be no association between breast cancer risk and active smoking either with or without additional passive smoking exposure. However, the use of such a reference group creates certain problems in analysing and interpreting results. In this study for example, never smoking women who were also not exposed to passive smoking were more likely to be nulliparous and recent users of oral contraceptives, two factors known to affect the risk of breast cancer (Collaborative Group on Hormonal Factors in Breast Cancer, 1996, 2002b).

There remains considerable controversy over the relationship between passive smoking and breast cancer since the results from previous studies are inconclusive; some suggested an increase in risk with exposure to passive tobacco smoke (Wells, 1991; Smith *et al*, 1994; Morabia *et al*, 1996; Millikan *et al*, 1998; Lash and Aschengrau, 1999; Delfino *et al*, 2000; Johnson *et al*, 2000; Liu *et al*, 2000; Kropp and Chang-Claude, 2002) and others showed no effect (Wartenberg *et al*, 2000; Nishino *et al*, 2001; Egan *et al*, 2002; Lash and Aschengrau, 2002; Gammon *et al*, 2004; Reynolds *et al*, 2004; Shrubsole *et al*, 2004; Bonner *et al*, 2005; Hanaoka *et al*, 2005; Lissowska *et al*, 2006). Recent reviews of the literature have also been inconsistent with two showing that there was an association between passive smoking and breast cancer (Johnson, 2005; Miller *et al*, 2007) and a further two suggesting that more evidence is needed before any conclusions can be drawn (IARC, 2004; US Surgeon General, 2006). The main reason for the difference in the reviews is that the latter two included all published studies, but in the other reviews various subanalyses were performed controlling for quality measures of the exposure assessment – specifically which aspects of passive smoking were assessed and what assessment method of passive smoking was used.

The two reviews that suggested there was an association between passive smoking and breast cancer also highlighted that the relationship appeared stronger for premenopausal compared with postmenopausal breast cancer (Johnson, 2005; Miller *et al*, 2007). However, only about half of the published studies provided results stratified by menopausal status (Wells, 1991; Millikan *et al*, 1998; Delfino *et al*, 2000; Johnson *et al*, 2000; Gammon *et al*, 2004; Reynolds *et al*, 2004; Shrubsole *et al*, 2004; Bonner *et al*, 2005; Hanaoka *et al*, 2005; Lissowska *et al*, 2006) and therefore the possibility of publication bias cannot be ruled out. The current study adds to the totality of the evidence suggesting that exposure to passive smoking in the home is not associated with an increase in the risk of breast cancer, and does not support the view that there is an increased risk in premenopausal women. We were not able to adequately examine the role of passive smoking in postmenopausal women, as over 90% of the women in this study were premenopausal and the majority (78%) of the peri/postmenopausal women were classified as such on the basis of their being current users of HRT.

As noted by previous reviews (Johnson, 2005; Miller *et al*, 2007), one of the difficulties in studies investigating passive tobacco smoking is both in the definition and measurement of the exposure variable. It has been suggested that adult exposure and childhood exposure could have different biological effects and should, therefore, be investigated separately (IARC, 2004; US Surgeon General, 2006). In some studies, like the current, the focus is solely on exposure from partners smoking within the home and did not quantify additional sources of exposure such as during childhood or from the workplace. This can lead to some misclassification of the exposure which is most likely to be nondifferential; however, given that over 50% of the study participants were exposed to passive smoking in the home, we would not expect there to be substantial misclassification. Nevertheless, such misclassification may have limited our ability to detect an association and we are unable to exclude a modest association between passive smoking and breast cancer.

One of the limitations of the current study is its retrospective nature leading to many possible biases in the assessment of the exposure of interest and relevant confounding variables. Women were asked about their smoking history, both active and passive, after the diagnosis of breast cancer, and it is possible that there are systematic differences in the reporting of responses between cases (who knew they had breast cancer) and controls. However, at the time this study was carried out, there was less public concern over the dangers of passive smoking and therefore less chance that such retrospective reporting bias would be an issue. Since this study was performed, the prevalence of smoking among women has fallen consistently year on year and the likelihood of exposure to passive smoke both in the workplace and the home has also fallen. However, neither of these changes will have any impact on the generalisability of the results of this study, since they only affect the prevalence of the exposure variable. It is possible that this study failed to recruit heavy persistent smokers who may have died prior to being eligible for this study; however, since it recruited people before the age of 45 years where tobacco-related death rates are low, this is unlikely to be a major source of bias. A strength of the study is that the data were collected during in-person interviews by a small number of trained nurses and is complete. Furthermore, the study obtained high-quality measurements of both the smoking and passive smoking exposure, with a full history of dates starting smoking, stopping smoking and numbers of cigarettes per day being recorded whenever significant changes occurred.

In summary, the current study finds no significant associations between either active smoking or passive smoking in the home and risk of breast cancer in women aged 36–45 years.

## Figures and Tables

**Table 1:** Descriptive characteristics of all cases and controls

**Characteristic**	**Cases (%), *n*=639**	**Controls (%), *n*=640**	***P*-value**
*Socioeconomic status*			
Professional	157 (25)	170 (27)	0.71
Nonmanual	219 (34)	212 (33)	
Manual/not employed	263 (41)	258 (40)	
			
*Use of oral contraceptives*			
Never	110 (17)	130 (20)	0.37
Last use <5 years ago	117 (18)	112 (18)	
Last use⩾5 years ago	412 (64)	398 (62)	
			
*Alcohol consumption*			
Non-drinkers	210 (33)	206 (32)	0.89
1–50 g week^−1^	261 (41)	270 (42)	
50+ g week^−1^	168 (26)	164 (26)	
			
*History of breast cancer in first-degree relative*			
No	557 (87)	585 (91)	0.02
Yes	71 (11)	43 (7)	
N/K	11 (2)	12 (2)	
			
Mean height (cm)	163.8	163.1	0.06
Mean weight (kg)	62.5	63.5	0.09
Mean BMI (kg m^−2^)	23.3	23.9	0.01
Mean age at menarche (years)	12.5	12.6	0.16
			
*Parity*			
Nulliparous	93 (15)	79 (12)	0.25
Parous	546 (85)	561 (88)	
			
Mean age at first giving birth (years) (among parous)	24.4	24.1	0.30
			
*Menopausal status*			
Premenopausal	591 (92)	595 (93)	0.74
Peri/postmenopausal	48 (8)	45 (7)	
			
*Active smoking exposure*			
Never	297 (46)	310 (48)	
Past	156 (24)	144 (23)	
Mean cigarettes per day	11.3	10.7	
Mean years smoked	10.8	10.6	
Mean age started smoking	17.8	17.7	
Current	186 (29)	186 (29)	
Mean cigarettes per day	14.0	14.1	
Mean years smoked	23.2	22.6	
Mean age started smoking	17.6	17.7	
			
*Active and passive exposure*			
Never smoker, no passive exposure	175 (27)	175 (27)	
Never smoker, passive exposure	122 (19)	135 (21)	
Mean years of exposure	12.2	13.3	
Past smoker, no passive exposure	58 (9)	52 (8)	
Past smoker, passive exposure	98 (15)	92 (14)	
Mean years of exposure	12.0	12.9	
Current smoker, no passive exposure	44 (7)	38 (6)	
Current smoker, passive exposure	142 (22)	148 (23)	
Mean years of exposure	16.2	14.8	

**Table 2:** Descriptive characteristics of controls by exposure to passive smoking among never smokers

**Characteristic**	**Neither active smoker nor exposed to passive smoking, *n*=175**	**Exposed to passive but not active smoking, *n*=135**	***P*-value**
*Socioeconomic status*			
Professional	48 (27)	27 (20)	0.30
Nonmanual	62 (35)	50 (37)	
Manual/not employed	65 (37)	58 (43)	
			
*Use of oral contraceptives*			
Never	51 (29)	27 (20)	0.01
Last use <5 years ago	36 (21)	17 (13)	
Last use ⩾5 years ago	88 (50)	91 (67)	
			
*Alcohol consumption*			
Non-drinkers	67 (38)	46 (34)	0.20
1–50 g week^−1^	71 (41)	68 (50)	
50+ g week^−1^	37 (21)	21 (16)	
			
*History of breast cancer in first-degree relative*			
No	161 (92)	122 (90)	0.62
Yes	10 (6)	11 (8)	
N/K	4 (2)	2 (1)	
			
Mean height (cm)	163.0	161.9	0.15
Mean weight (kg)	64.9	62.6	0.09
Mean BMI (kg m^−2^)	24.5	23.4	0.28
Mean age at menarche (years)	12.5	12.7	0.24
			
*Parity*			
Nulliparous	31 (18)	12 (9)	0.03
Parous	144 (82)	123 (91)	
			
Mean age at first giving birth (years) (among parous)	25.3	24.5	0.15
			
*Menopausal status*			
Premenopausal	167 (95)	126 (93)	0.42
Peri/postmenopausal	8 (5)	9 (7)	

**Table 3:** Relative risks of breast cancer by active and passive smoking

			**Stratified by age and region**	**Additionally adjusted^a^**	**Additionally and mutually adjusted^b^**
**Exposure**	**Cases**	**Controls**	**RR (95% CI)**	**RR (95% CI)**	**RR (95% CI)**
*Active smoking exposure*					
Never smoker	297	310	1.00	1.00	
Past smoker	156	144	1.13 (0.86–1.49)	1.15 (0.87–1.53)	1.00
Current smoker	186	186	1.04 (0.80–1.35)	1.04 (0.79–1.36)	0.90 (0.54–1.50)
					
*Intensity (per 5 cigarettes per day)*					
Ever smoker	342	330	1.02 (0.91–1.13)	1.00 (0.89–1.12)	1.00 (0.88–1.12)
					
*Duration (per 5 years smoked)*					
Ever smoker	342	330	1.02 (0.93–1.12)	1.00 (0.91–1.11)	1.02 (0.86–1.22)
					
*Age started smoking (per 5 years)*					
Ever smoker	342	330	0.97 (0.80–1.19)	0.97 (0.79–1.19)	0.99 (0.76–1.28)
					
*Active and passive exposure*					
Never smoker, no passive exposure	175	175	1.00	1.00	
Never smoker, passive exposure	122	135	0.90 (0.65–1.25)	0.89 (0.64–1.25)	
					
Past smoker, no passive exposure	58	52	1.12 (0.73–1.72)	1.12 (0.72–1.73)	
Past smoker, passive exposure	98	92	1.06 (0.75–1.51)	1.09 (0.75–1.56)	
					
Current smoker, no passive exposure	44	38	1.15 (0.71–1.87)	1.19 (0.72–1.95)	
Current smoker, passive exposure	142	148	0.96 (0.70–1.31)	0.93 (0.67–1.30)	
					
*Number of years of exposure to passive smoking at home*					
Never smoker, 0 years	175	175	1.00	1.00	
Never smoker, 1–10 years	56	55	1.02 (0.66–1.56)	0.99 (0.64–1.53)	
Never smoker, 11+ years	66	80	0.83 (0.56–1.22)	0.84 (0.56–1.25)	
				*P*-value^c^=0.31	
					
Past smoker, 0 years	58	52	1.12 (0.73–1.72)	1.12 (0.72–1.73)	
Past smoker, 1–10 years	42	38	1.10 (0.68–1.79)	1.12 (0.68–1.85)	
Past smoker, 11+ years	56	54	1.04 (0.68–1.59)	1.06 (0.68–1.66)	
				*P*-value^c^=0.71	
					
Current smoker, 0 years	44	38	1.15 (0.71–1.87)	1.19 (0.72–1.96)	
Current smoker, 1–10 years	31	44	0.71 (0.43–1.17)	0.66 (0.39–1.10)	
Current smoker, 11+ years	111	104	1.07 (0.76–1.50)	1.06 (0.74–1.53)	
				*P*-value^c^=0.58	

a Additionally adjusted for socioeconomic status, alcohol consumption, BMI, parity and age at first giving birth, use of oral contraceptives, family history of breast cancer, age at menarche, menopausal status.

b Adjustments as for (a), but with mutual adjustment for all active smoking-related variables among the ever smokers.

c *P*-value for linear trend test of increasing duration of passive smoking exposure.

**Table 4:** Relative risks of breast cancer by passive smoking exposure among never smokers by different characteristics

	**Cases**	**Controls**	
**Subgroup**	**Exposed/unexposed**		**RR (95% CI)^a^**
*Menopausal status*			
Premenopausal	112/170	126/167	0.83 (0.59–1.17)
Peri/postmenopausal	7/4	7/5	1.51 (0.19–12.2)
			*P*-value^b^=0.35
			
*Alcohol consumption*			
Never drinkers	42/66	42/64	0.93 (0.51–1.69)
Drinkers	79/107	89/108	0.86 (0.56–1.30)
			*P*-value=0.98
			
*Oral contraceptive use*			
Never user	11/35	17/39	0.68 (0.25–1.91)
Last use within 5 years	19/22	12/30	2.51 (0.90–6.99)
Last use greater than 5 years ago	85/111	91/88	0.74 (0.49–1.12)
			*P*-value=0.11
			
*Family history of breast cancer in mother or sister*			
No	108/154	122/161	0.89 (0.62–1.26)
Yes	8/9	9/7	1.12 (0.20–6.41)
			*P*-value=0.57
			
*Parity and age at first giving birth*			
Nulliparous	10/33	11/30	0.64 (0.21–1.91)
Parous and age at first giving birth <25 years	66/54	66/68	1.06 (0.63–1.78)
Parous and age at first giving birth 25+ years	45/78	57/74	0.68 (0.40–1.16)
			*P*-value=0.21
			
*Socioeconomic status*			
Professional	25/54	26/47	0.81 (0.40–1.63)
Nonmanual	40/55	50/61	0.80 (0.45–1.43)
Manual/not employed	50/52	57/61	1.03 (0.58–1.85)
			*P*-value=0.82
			
*Body mass index (kg m*^−*2*^)			
<25	89/137	95/116	0.72 (0.48–1.07)
25+	31/38	36/51	1.07 (0.54–2.14)
			*P*-value=0.18
			
*Age at menarche (years)*			
<12	61/82	56/81	1.09 (0.66–1.79)
13+	59/90	74/86	0.67 (0.42–1.09)
			*P*-value=0.15

a Relative risks are stratified by age and region and additionally adjusted for parity and oral contraceptive use.

b *P*-values are for tests of heterogeneity in effect between subgroup characteristics.
